# Impact of area socioeconomic deprivation on major adverse renal events in patients with acute kidney injury: a retrospective cohort study of a single-center national patient population

**DOI:** 10.1080/07853890.2026.2674449

**Published:** 2026-06-04

**Authors:** Xiang Yu, XinYan Gong, BaoLong Wang, YuWei Ji, SaSa Nie, RiLiGe Wu, WanLing Wang, MengJie Huang, GuangYan Cai, Zhe Feng

**Affiliations:** a Department of Nephrology, the First Medical Center of Chinese PLA General Hospital, Chinese PLA Institute of Nephrology, National Key Laboratory of Kidney Diseases, National Clinical Research Center for Kidney Diseases, Beijing Key Laboratory of Kidney Diseases Research, Beijing, China; b Medical Innovation Research Division, Chinese PLA General Hospital, Beijing, China

**Keywords:** Acute kidney injury, socioeconomic deprivation, major adverse renal events, health equity, cohort study

## Abstract

**Background:**

To investigate the impact of area socioeconomic deprivation on the risk of major adverse kidney events (MAKEs) within 90 days post-discharge among patients with acute kidney injury (AKI).

**Methods:**

This retrospective cohort study included patients with AKI between January 1, 2014, and December 31, 2023. Area socioeconomic deprivation index was calculated using principal component analysis based on district-level economic and healthcare resource data from patients’ registered residential areas. Patients were stratified into four deprivation quartiles (Q1–Q4) and analyzed with multivariable Cox proportional hazards regression models to assess the association between deprivation index and MAKE risk.

**Results:**

Among 5,934 AKI patients, 1,107 (18.7%) experienced MAKEs within 90-day follow-up. Multivariable Cox regression analysis revealed that patients in the highest deprivation quartile (Q4) had a significantly greater risk of MAKEs than those in the lowest deprivation quartile (Q1) (hazard ratio [HR] 1.48, 95% CI: 1.24–1.77), with a significant dose–response relationship between MAKE risk and (trend *p* < 0.001). Subgroup analysis revealed that the negative effect of area socioeconomic deprivation was more pronounced among patients with lower levels of health insurance coverage (resident medical insurance/Self-pay) (interaction *p* = 0.032). Mediation analysis indicated that the delay between discharge and the first outpatient follow-up mediated approximately 23.2% of the area socioeconomic deprivation effect.

**Conclusion:**

Area socioeconomic deprivation is a key socioenvironmental determinant of outcome in AKI patients. This finding suggests a significant association of macrolevel social environments on disease outcomes. Therefore, social environment assessments should be integrated into AKI clinical management systems for systematically reducing health inequalities.

## 1. Introduction

Acute kidney injury (AKI) is a major global public health threat, affecting 10–15% of hospitalized patients, and is associated with markedly elevated short-term mortality [1–3]. More critically, in the long term, AKI survivors face certain health risks, with approximately 15–20% of patients potentially developing chronic kidney disease post-discharge. Among these patients, 5% may progress to end-stage renal disease and thus require life-sustaining renal replacement therapy [4–6]. The substantial healthcare burden and socioeconomic costs associated with this disease continuum underscore the value of investigating factors that may be associated with disease progression in these patients.

In recent years, a greater understanding of the social determinants of health has led researchers to recognize that individual health outcomes are not only influenced by clinical factors but also closely linked to socioeconomic environments. Studies indicate that area socioeconomic deprivation—the systemic disadvantage of residents in specific geographic areas in terms of access to social resources, economic opportunities, and essential services—is significantly associated with the incidence and mortality rates of multiple chronic diseases, including cardiovascular disease and diabetes [7–10]. However, research on the impact of area socioeconomic factors on long-term patient outcomes in the field of AKI remains underexplored. Existing studies are largely limited to single regions, fail to adequately control for variations in clinical practice across healthcare institutions, and suffer from small sample sizes. These limitations result in substantial heterogeneity in the findings, making it difficult to generate high-level evidence [11,12].

To address this critical knowledge gap, this study leverages our hospital’s unique position as a medical center that treats patients nationwide to establish a large-scale national cohort. By implementing uniform, high-standard clinical protocols, this study effectively controlled for healthcare facility-level confounders, providing an ideal platform to examine the independent impact of area socioeconomic deprivation on AKI outcomes. This study aims to explore the dose–response relationship between area socioeconomic deprivation and major adverse kidney events (MAKEs) in AKI patients. The findings are expected to provide evidence-based support for identifying high-risk populations, establishing targeted post-discharge follow-up mechanisms, and formulating health policies aimed at narrowing health disparities.

## 2. Methods

### 2.1. Study design and population

This single-center retrospective cohort study initially screened all adult patients admitted to the First Medical Center of the Chinese PLA General Hospital between January 1, 2014, and December 31, 2023 (*N* = 84,011). As a national tertiary referral center, it draws patients from across the country. This patient flow provides a geographically diverse, national sample, while the single-center setting itself ensures uniformity in clinical protocols and data collection, thereby controlling for potential confounding arising from inter-hospital variations. From this source population, we identified 7,477 patients with AKI. The inclusion criteria for the final analytic cohort were as follows: ① age ≥18 years, ② hospitalization duration >48 h, ③ ≥2 serum creatinine measurements during hospitalization, and ④ complete medical records and follow-up data. The exclusion criteria included the opposite of the inclusion criteria and the following: ① prior diagnosis of chronic kidney disease (CKD; stage 3 or higher), as advanced CKD is a dominant risk factor for MAKEs and its inclusion could profoundly confound the association between area socioeconomic deprivation and outcomes, ② maintenance hemodialysis, peritoneal dialysis, or kidney transplantation, and ③ missing key clinical variables or household registration information. After applying the inclusion and exclusion criteria, 5,934 patients with AKI constituted the final study cohort. These patients originated from 2,176 unique counties across China, demonstrating a wide geographic distribution. This study strictly adhered to the ethical principles of the Declaration of Helsinki and was approved by the Ethics Review Committee of the PLA General Hospital (Approval No.: Lunzi No. S2025-260-01). The Ethics Review Committee waived the requirement for informed consent in accordance with the “Measures for the Ethical Review of Life Science and Medical Research Involving Humans”, as the research was retrospective and utilized exclusively anonymized data.

### 2.2. AKI case identification and onset date determination

We relied on the PLA General Hospital Big Data Center for AKI patient data identification and extraction. All AKI diagnoses adhered to the 2020 Kidney Disease: Improving Global Outcomes (KDIGO) guidelines, according to which AKI was defined as either an increase in the serum creatinine level of 1.5 times the baseline value during hospitalization or an absolute increase in the serum creatinine level exceeding 26.5umol/L (0.3 mg/dl) within 48 h [13] For patients with community-acquired AKI, the onset time was defined as the earliest recent diagnosis date for emergency and outpatient admissions. For referred patients, owing to differences in prehospital care information, the onset time was defined as the admission date. Patients with hospital-acquired AKI were identified *via* structured query language scripts based on the diagnostic criteria and filtered from Oracle Database 10 g. The implementation of the diagnostic criteria was conducted in accordance with the following principles. First, all serum creatinine measurements during hospitalization were arranged chronologically. The system time of the first valid measurement was designated t_1_, the second was t_2_ (where t_2_ - t_1_≤ 7 days), and so forth. Any KDIGO-compliant serum creatinine level change occurring within the interval t_n_ - t_n+1_ was identified as an AKI event. The 24-hour period starting from t_n+1_ was designated as the AKI onset time to minimize exposure misclassification bias. Owing to the uncertainty in recording the 24-hour fluid balance outside the ICU and the instability of the available data, the urine output criterion was not adopted in this study. A flowchart detailing the AKI identification algorithm is provided in Supplementary Figure S1.

To operationally distinguish community-acquired from hospital-acquired AKI, particularly for patients without a documented pre-admission serum creatinine, we employed an algorithm adapted from the epidemiological method described by Xu et al. in 2015 [14]. An AKI episode was classified as community-acquired if the patient met at least one of the following criteria at hospital presentation: ① a documented admission diagnosis of AKI; ② a serum creatinine change on the first hospital day meeting the KDIGO definition for AKI; ③ an elevated admission creatinine, defined as ≥1.4 mg/dL for men or ≥ 1.1 mg/dL for women, which was also at least 1.5 times higher than the lowest creatinine value recorded during the subsequent hospitalization. All other AKI episodes identified during the hospitalization, which did not meet these criteria for community onset, were classified as hospital-acquired.

### 2.3. Definition and follow-up of major adverse kidney events

The primary outcome was MAKEs occurring within 90 days post-discharge, defined as a composite endpoint occurring after index discharge that included any of the following: ① all-cause mortality, ② new dialysis dependency (receiving renal replacement therapy for >30 days or kidney transplantation), and ③ persistent renal function loss (estimated glomerular filtration rate (eGFR) decline ≥40% from baseline). Secondary outcomes included the 90-day all-cause readmission rate and failure to regain baseline renal function at discharge (defined as serum creatinine not returning to within 1.5 times baseline levels). Patient follow-up data were retrieved from the hospital’s electronic medical record system with a follow-up cutoff date of December 31, 2024. Patient mortality status was determined through data linkage with the Chinese Center for Disease Control and Prevention (China CDC), and the ID number, name, sex, and date of birth were used as identifiers. Patients with no matching information were considered alive. Patients with no follow-up records in the hospital’s electronic medical record system after discharge were considered lost to follow-up, and their data were truncated at 90 days post-index discharge.

### 2.4. Definition of covariates and data collection

To control for potential confounding biases, we extracted the following covariates from the electronic medical records for multivariable model adjustment: demographic characteristics, socioeconomic characteristics, baseline health status, renal function status at admission, AKI severity, and hospitalization status. Demographic data encompassed age, sex, and BMI. Socioeconomic data included the occupational category and health insurance type. The health insurance type, which served as a key proxy for individual socioeconomic status, was grouped into a dichotomous variable based on the coverage level: employee medical insurance/government-sponsored insurance versus resident medical insurance/self-pay. Employee medical insurance and government-sponsored insurance in China typically provide more comprehensive coverage, with higher reimbursement rates and lower out-of-pocket costs for patients. In contrast, resident medical insurance offers more basic coverage with lower reimbursement ceilings, and self-pay indicates no insurance coverage. The occupational category, another indicator of individual socioeconomic status, was consolidated from original occupational data into a three-category variable based on the nature of the job: nonmanual occupations, manual occupations, and no stable occupation/retired. Nonmanual occupations included roles such as managers, professionals, technicians, and clerical staff. Manual occupations encompassed workers in agriculture, industry, and service sectors primarily engaged in physical labor. This combined category groups individuals who are not in formal, current employment, a practical classification that distinguishes them from the actively working population in our study setting. Baseline health status was quantified *via* the Charlson comorbidity index to assess the burden of comorbidities. Calculated according to the revised criteria of Quan et al. it included hypertension, diabetes, cardiovascular and cerebrovascular diseases, chronic pulmonary diseases, tumors (nonadvanced and without metastasis), and cirrhosis (with complications), and acute complications related to AKI [15] were excluded. Renal function status at admission was assessed *via* the estimated glomerular filtration rate (eGFR) calculated from the initial serum creatinine level upon admission according to the CKD-EPI (2021) formula. This metric reflects the patient’s immediate renal function level at presentation and is influenced by both baseline renal function and the severity of acute injury [16]. AKI severity was primarily classified *via* the KDIGO staging system. Hospitalization status included ICU admission and major surgery. Post-discharge medication adherence and health literacy data were excluded from the model adjustments because of incomplete availability in the electronic medical records.

### 2.5. Statistical analysis methods

#### 2.5.1. Development of the area socioeconomic deprivation index

The area socioeconomic deprivation index quantifies socioeconomic disadvantage at the county level. In this study, administrative district codes were extracted from the first six digits of patient ID numbers and precisely matched with corresponding county codes from the China County Statistical Yearbook and China Health Statistics Yearbook for the same period. Owing to data availability constraints, this study prioritized core indicators that could be obtained continuously at the county level. Dimensions such as education level and employment opportunities were excluded because county-level data for certain years were missing. Ultimately, five indicators were selected: economic dimensions (per capita GDP, per capita disposable income, and general public budget revenue) and healthcare resource dimensions (number of licensed physicians per thousand population and number of healthcare institution beds per thousand population). Data collection spanned from January 1, 2014, to December 31, 2023. Missing data across counties for each year were verified. For indicators with a missing rate <5%, data from the preceding year were used for imputation. Consistency checks were performed on the imputed data by comparing the mean, median, and standard deviation of each indicator before and after imputation. The results revealed no statistically significant differences (*p* > 0.05), indicating that the imputation method had no significant effect on data quality. Counties with a missing rate ≥5% were excluded.

This index was constructed specifically for this study. While it is not a direct application of a pre-existing index, its development followed the established methodological paradigm of applying principal component analysis to multidimensional area-level data, aligning with the conceptual framework of composite deprivation measures. To ensure consistent units of measurement, the raw data were subjected to Z score normalization. Principal component analysis (PCA) was applied to reduce the dimensionality of the five normalized indicators. The Kaiser–Meyer–Olkin test and Bartlett’s test of sphericity confirmed the suitability of PCA. The first principal component with an eigenvalue greater than 1 was extracted. Comprehensive scores for each district were calculated based on its score coefficient matrix, where higher scores indicate lower socioeconomic deprivation. This principal component had an eigenvalue of 3.27 and contributed 65.3% of the variance, demonstrating its ability to effectively summarize the original indicator information. All indicator loadings were relatively high (range: 0.71–0.92), as detailed in Supplementary Table S1. For analytical convenience, patients were grouped into four quartiles based on composite scores: Q1, Q2, Q3, and Q4. Group Q4 exhibited the highest deprivation level, whereas Q1 represented the lowest.

#### 2.5.2. Correlation analysis

Kaplan–Meier curves depicting the cumulative incidence of MAKEs were plotted for the four patient groups (Q1–Q4), and the log-rank test was used to assess intergroup differences. The hazard ratios for the occurrence of MAKEs across different area socioeconomic deprivation groups and their 95% confidence intervals were calculated. Three models were established: Model 1 adjusted for only age and sex; Model 2 further adjusted for individual-level socioeconomic indicators, including health insurance type and occupational category, and was constructed with the aim of assessing how area socioeconomic deprivation effects change after controlling for individual socioeconomic differences.Model 3 was the fully adjusted model designed to minimize confounding by clinical severity and comorbidity burden. In addition to all covariates from Model 2, it explicitly adjusted for the Charlson comorbidity index, admission eGFR, AKI stage, and ICU admission status. The Charlson index serves as a composite measure of key comorbidities, and its inclusion along with ICU status directly addresses adjustment for illness severity. This model provides the final estimate of the independent association between area socioeconomic deprivation and MAKEs. Additionally, area socioeconomic deprivation index grouping was incorporated as a continuous variable to test for trends, and the P value for trend was calculated. The proportional hazards assumption for the Cox models was assessed both graphically (by inspecting log-minus-log plots) and formally using the Schoenfeld residual test. No significant violations of the proportionality assumption were detected for the primary exposure variable (area socioeconomic deprivation quartile) or for the models as a whole (all *p* > 0.05), supporting the appropriateness of the Cox model. Prior to constructing the multivariable Cox regression model, variance inflation factors (VIFs) were calculated for all continuous variables to assess multicollinearity. All VIFs were less than 5, indicating that any multicollinearity would not significantly compromise the stability of the model parameters.

#### 2.5.3. Subgroup analysis and interaction testing

To examine the robustness and generalizability of the association between area socioeconomic deprivation and MAKEs, we conducted analyses in predefined subgroups. Subgroup variables included age (<65 years/≥65 years), gender, renal function at admission (eGFR < 90 or ≥ 90 mL/min/1.73 m^2^), AKI severity (KDIGO stages 1/2/3), and the AKI type (community acquired/hospital acquired). Additionally, to explore health equity issues, we specifically examined whether the effect of area socioeconomic deprivation differed across individuals with varying socioeconomic statuses, with health insurance type serving as the key subgroup variable. Subgroup association analyses employed multivariable Cox proportional hazards models. For each subgroup, the models adjusted for major confounders, excluding the subgroup stratification variable. Likelihood ratio tests with interaction terms were performed to assess whether area socioeconomic deprivation effects differed across subgroups.

#### 2.5.4. Mediator analysis

To explore the potential mechanisms underlying the impact of area socioeconomic deprivation on long-term outcomes in AKI patients, a regression-based causal mediation analysis within the counterfactual framework was performed. It was hypothesized that area socioeconomic deprivation partially increases the risk of MAKEs by prolonging the time from hospital discharge to the first outpatient follow-up visit. The mediating variable “time to first post-discharge follow-up” was defined as the number of days from the index discharge date to the earliest date of any outpatient visit recorded in the hospital’s electronic medical record system. For patients with no outpatient records at the hospital from discharge until the follow-up cutoff date, their data were administratively censored at 90 days post-discharge. The analysis involved fitting separate models for the mediator and the outcome, adjusting for baseline confounders including age, sex, and the Charlson comorbidity index. Confidence intervals for the direct and indirect effects were calculated *via* nonparametric bootstrapping with 1,000 resamples to ensure robustness. The magnitude of the mediating effect was expressed as the proportion of the total effect attributable to mediation.

#### 2.5.5. Sensitivity analysis

To assess the robustness of the primary findings, two sensitivity analyses were prespecified. Method 1 involved altering the outcome measure by using the difference in restricted mean survival as a supplementary analysis. The mean difference in survival time free of MAKEs between the highest deprivation group (Q4) and the lowest deprivation group (Q1) during the 90-day follow-up period, along with its 95% confidence interval, was calculated. Method 2 modified the exposure definition by incorporating the area socioeconomic deprivation index as a continuous variable into a multivariable Cox proportional hazards model. The hazard ratio and its 95% confidence interval were calculated for each additional standard deviation of the index to examine its continuous dose–response relationship with MAKE risk.

**Figure 1.**
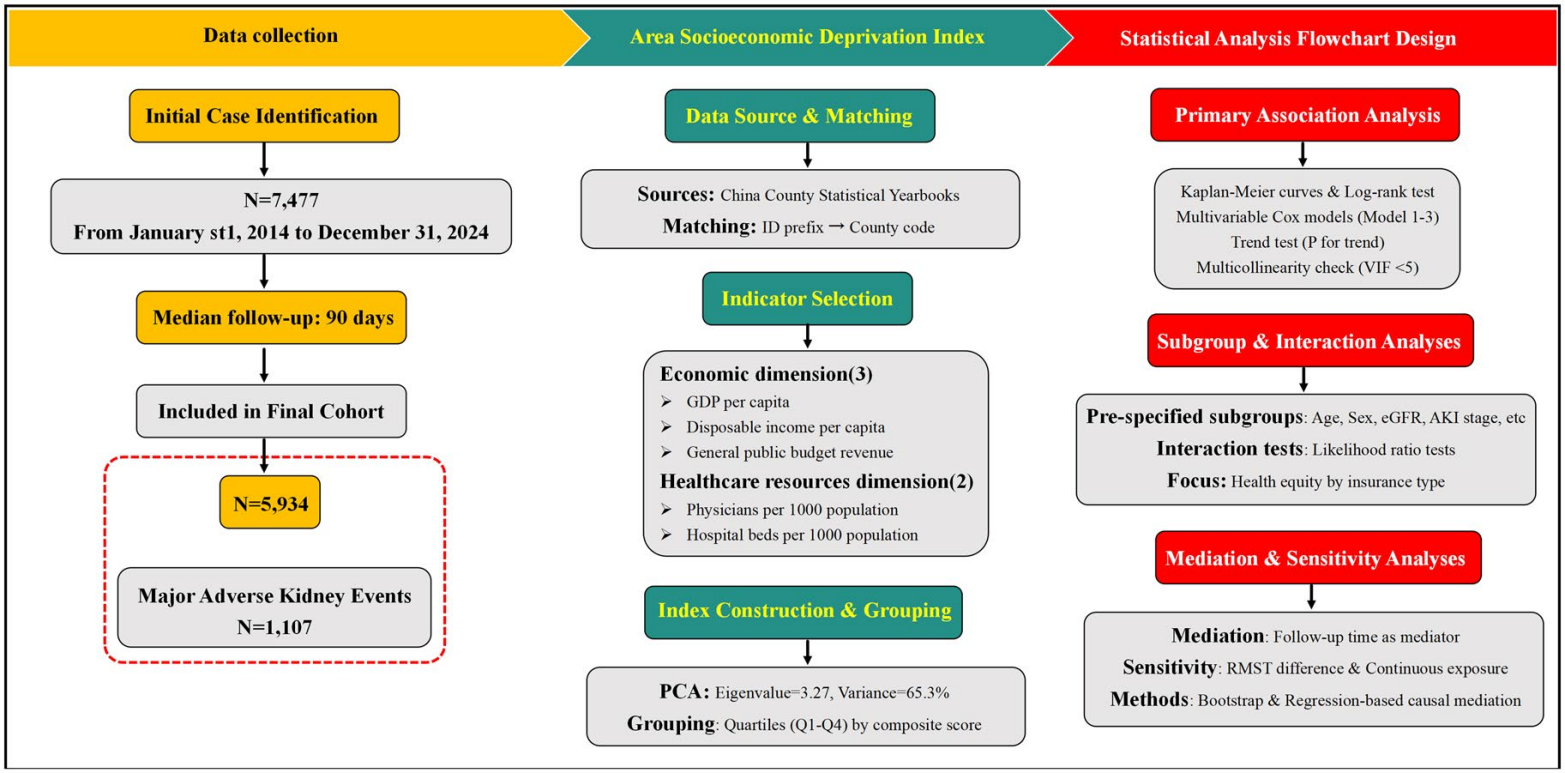
Study flowchart. Note: The cohort consisted of 5,934 AKI patients from an initial 7,477 cases (2014–2024). The Area Socioeconomic Deprivation Index was constructed using five county-level indicators via Principal Component Analysis and categorized into quartiles (Q1-Q4). Statistical analyses included primary association, subgroup/interaction, and mediation/sensitivity analyses.

All data were entered with Oracle Database 10 g and saved in CSV format. The study workflow is illustrated in Figure 1. Data reading and modeling were performed with the R-Studio statistical software package (version 4.1.0, R Foundation for Statistical Computing, Vienna, Austria), particularly the survival, survRM2, and mediation packages. Custom coding was employed for subsequent computational processing; each variable was associated with marked time and type, and clinical data were matched with district-level macroeconomic data. All p values were two-tailed, with values <0.05 considered to indicate statistical significance.

## 3. Results

### 3.1. Area socioeconomic deprivation stratification and population baseline characteristics

The area socioeconomic deprivation index, constructed from county-level socioeconomic and healthcare resource data, is greater for lower levels of deprivation. Patients were stratified into four groups based on quartiles of this index: Q1 (lowest deprivation, index range >0.85), Q2 (index range −0.41 to 0.85), Q3 (index range −1.52 to −0.41), and Q4 (highest deprivation, index range ≤-1.52).

Patient characteristics exhibited distinct distribution patterns across deprivation groups. Among Q4 patients with the highest area socioeconomic deprivation indices, the proportion covered by employee medical insurance/government-sponsored insurance (36.3%) was lower than that in Q1 (54.0%) (*p* < 0.001), and the proportion engaged in nonmanual occupations (32.0%) was also lower than that in Q1 (47.2%) (*p* < 0.001). Additionally, patients in the Q4 group had higher rates of diabetes and cardiovascular/cerebrovascular diseases, along with a higher median Charlson comorbidity index score (*p* < 0.001). However, the distribution of patients across groups was similar in terms of age, sex, BMI, the proportion of RRT, the proportion of ICU admission, and other comorbidities. Although the proportion of stage 3 AKI patients showed an increasing trend with increasing area socioeconomic deprivation, there were no statistically significant differences between groups (*p* = 0.886) (Table 1).

**Table 1.** Baseline characteristics of patients by area socioeconomic deprivation quartiles.

Patient Characteristic	All Patients (*n* = 5934)	Q1 Group (*n* = 1484)	Q2 Group (*n* = 1483)	Q3 Group (*n* = 1484)	Q4 Group (*n* = 1483)	P value
**Demographics**						
Age (years), Mean ± SD	65.2 ± 12.3	64.5 ± 11.8	65.0 ± 12.2	65.8 ± 12.7	66.1 ± 12.9	0.084
Male, n (%)	3708 (62.5)	926 (62.4)	931 (62.8)	927 (62.5)	924 (62.3)	0.991
BMI (kg/m²), Mean ± SD	24.5 ± 3.8	24.3 ± 3.6	24.6 ± 3.7	24.7 ± 3.9	24.4 ± 4.0	0.745
**Socioeconomic Factors**						
Health Insurance Type (employee medical insurance/government-sponsored insurance), n (%)	2540 (42.8)	801 (54.0)	652 (44.0)	548 (36.9)	539 (36.3)	<0.001
Occupational Category, n (%)						<0.001
Nonmanual	2210 (37.2)	701 (47.2)	552 (37.2)	482 (32.5)	475 (32.0)	
Manual	2450 (41.3)	551 (37.1)	601 (40.5)	651 (43.9)	647 (43.6)	
Unstable/Retired	1274 (21.5)	232 (15.6)	330 (22.3)	351 (23.7)	361 (24.3)	
**Baseline Health Status**						
Charlson Comorbidity Index, Median (IQR)	4 (2–6)	3 (2–5)	4 (2–6)	4 (2–6)	5 (3–7)	<0.001
Hypertension, n (%)	3560 (60.0)	848 (57.1)	882 (59.5)	912 (61.5)	918 (61.9)	0.045
Diabetes, n (%)	2134 (36.0)	448 (30.2)	521 (35.1)	562 (37.9)	603 (40.7)	<0.001
Cardiovascular Disease, n (%)	1780 (30.0)	398 (26.8)	432 (29.1)	462 (31.1)	488 (32.9)	0.002
Chronic Pulmonary Disease, n (%)	1187 (20.0)	269 (18.1)	292 (19.7)	311 (21.0)	315 (21.2)	0.088
Malignancy, n (%)	891 (15.0)	198 (13.3)	222 (15.0)	231 (15.6)	240 (16.2)	0.105
Liver Cirrhosis, n (%)	297 (5.0)	64 (4.3)	71 (4.8)	79 (5.3)	83 (5.6)	0.215
**Clinical Status at Admission**						
Admission eGFR (mL/min/1.73 m²), Mean ± SD	45.2 ± 25.8	46.0 ± 25.2	45.5 ± 25.7	44.8 ± 26.0	44.2 ± 26.3	0.255
AKI Stage, n (%)						0.886
Stage 1	2967 (50.0)	752 (50.7)	745 (50.2)	738 (49.7)	732 (49.4)	
Stage 2	1780 (30.0)	443 (29.9)	444 (29.9)	446 (30.1)	447 (30.1)	
Stage 3	1187 (20.0)	289 (19.5)	294 (19.8)	300 (20.2)	304 (20.5)	
**In-Hospital Management**						
RRT, n (%)	1187 (20.0)	294 (19.8)	298 (20.1)	299 (20.2)	296 (20.0)	0.998
ICU Admission, n (%)	2967 (50.0)	743 (50.1)	740 (49.9)	743 (50.1)	741 (50.0)	1.000

**Abbreviations:** AKI, acute kidney injury; BMI, body mass index; eGFR, estimated glomerular filtration rate; ICU, intensive care unit; IQR, interquartile range; RRT, renal replacement therapy; SD, standard deviation.

### 3.2. Association between area socioeconomic deprivation and MAKEs

**Figure 2.**
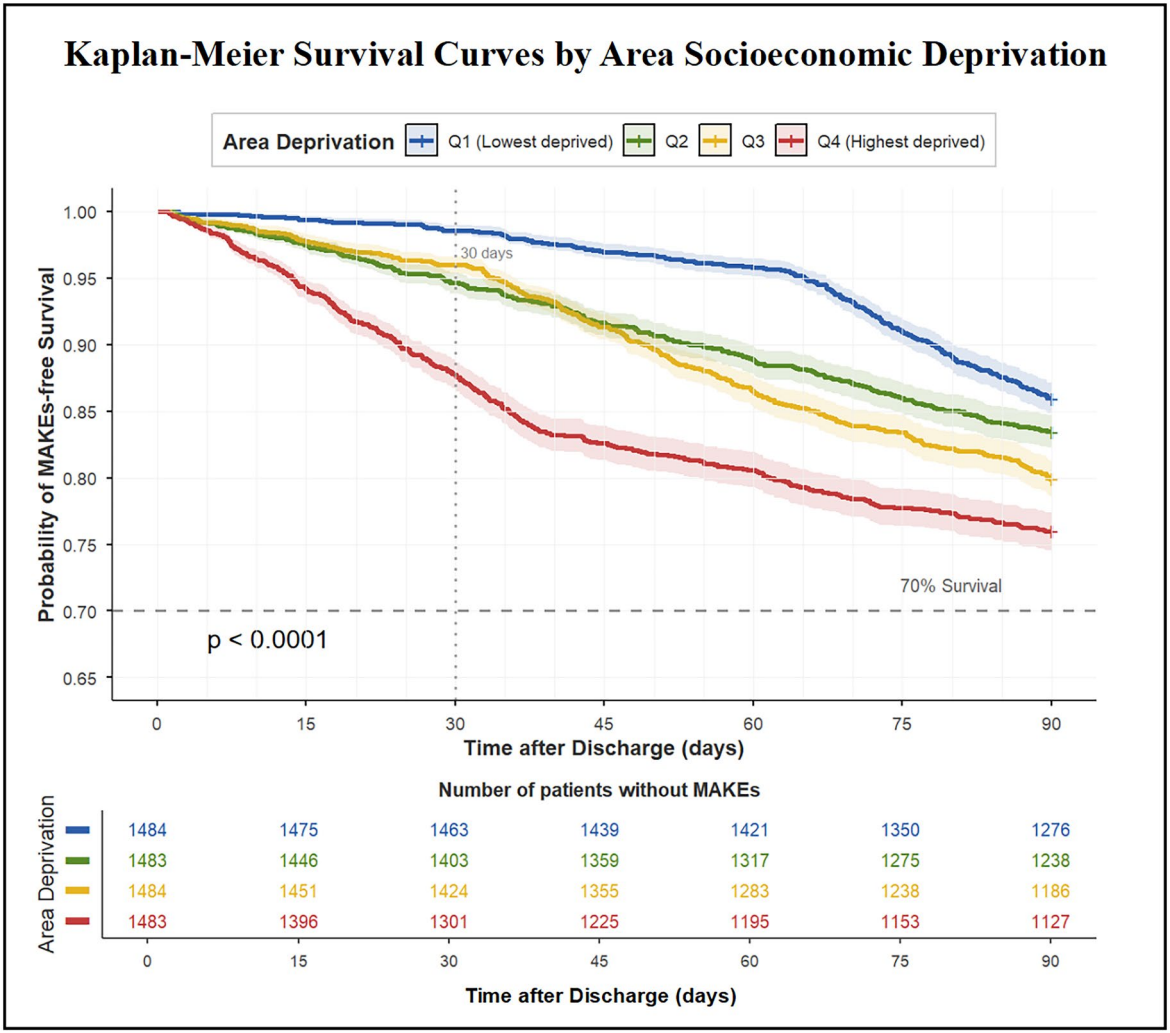
Kaplan-Meier curves depicting 90-day MAKEs-free survival stratified by area socioeconomic deprivation quartiles. Note: The figure presents the cumulative probability of survival without Major Adverse Kidney Events (MAKEs) over 90 days following hospital discharge. Patients are stratified into four groups (Q1-Q4) based on the Area Socioeconomic Deprivation Index, with Q1 representing the least deprived areas and Q4 the most deprived. The survival curves demonstrate a clear, graded separation from early follow-up, with the most deprived group (Q4) consistently exhibiting the lowest probability of MAKEs-free survival. A dashed vertical reference line marks the 30-day time point, and a horizontal line indicates the 70% survival probability threshold. The highly significant log-rank p-value (< 0.0001) confirms a statistically significant difference in outcomes across deprivation levels. The table beneath the plot details the number of patients who remained event-free and were still under observation (at risk) at 0, 30, 60, and 90 days for each quartile.

Within the 90-day follow-up periods, 1,107 patients (18.7%) experienced MAKEs. Kaplan–Meier survival curves revealed a significant decrease in the cumulative MAKE-free survival rate with increasing area socioeconomic deprivation (log-rank test *p* < 0.001; Figure 2). Multivariable Cox proportional hazards regression analysis indicated that area socioeconomic deprivation was independently associated with an increased risk of 90-day MAKEs. In Model 1, compared with the lowest deprivation quartile (Q1), the highest deprivation quartile (Q4) exhibited an 80% increased risk of MAKEs (HR 1.80, 95% CI 1.51–2.15), with a significant dose–response relationship (P for trend < 0.001). In Model 2, the Q4 group’s HR decreased to 1.65 (95% CI 1.38–1.97). In Model 3, which was further adjusted for all potential confounders, the association between area socioeconomic deprivation and MAKEs remained significant and independent. The risk of MAKEs in the Q4 group was still 1.48 times that in the Q1 group (95% CI 1.24–1.77), and the trend test remained significant (P for trend < 0.001) (Table 2).

**Table 2.** Multivariable associations between area socioeconomic deprivation quartile and the risk of MAKEs.

Area Socioeconomic Deprivation Group	No. of Patients (Events)	Model 1: HR (95% CI)	Model 2: HR (95% CI)	Model 3: HR (95% CI)
Q1 (Least deprived)	1484 (208)	1.00 (Reference)	1.00 (Reference)	1.00 (Reference)
Q2	1483 (245)	1.18 (0.98–1.42)	1.15 (0.96–1.38)	1.12 (0.93–1.35)
Q3	1484 (298)	1.45 (1.21–1.74)	1.36 (1.14–1.63)	1.28 (1.07–1.53)
Q4 (Most deprived)	1483 (356)	1.80 (1.51–2.15)	1.65 (1.38–1.97)	1.48 (1.24–1.77)
P for trend		<0.001	<0.001	<0.001

**Abbreviations:** MAKEs, Major Adverse Kidney Events; HR, hazard ratio; CI, confidence interval; eGFR, estimated glomerular filtration rate; AKI, acute kidney injury; ICU, intensive care unit; VIF, variance inflation factor.

**Note:** Model 1: Adjusted for age and sex. Model 2: Adjusted for Model 1 covariates plus individual-level socioeconomic factors (health insurance type and occupational category). Model 3: Fully adjusted model, incorporating all covariates from Model 2 plus the Charlson Comorbidity Index, baseline eGFR, AKI stage, and ICU admission status.

### 3.3. Subgroup analysis and interactions

Subgroup analyses revealed that the association between area socioeconomic deprivation and increased MAKE risk was consistent across most prespecified subgroups, demonstrating the same directionality as that in the primary analysis (i.e. HR > 1). No significant effect modification was observed in the subgroups stratified by age, sex, baseline renal function, AKI severity, or AKI type (P for interaction > 0.05), indicating that this association was broadly applicable across patient populations with different clinical characteristics. However, a significant interaction in the critical insurance type subgroup was detected (P for interaction = 0.032). Specifically, the negative effect of area socioeconomic deprivation was more pronounced among patients with lower levels of medical coverage (resident medical insurance/self-pay; HR 1.63, 95% CI 1.33–2.00), whereas among employee medical insurance/government-sponsored insurance-funded patients with higher coverage, the association showed an increasing trend but remained nonsignificant (HR 1.25, 95% CI 0.95–1.64) (Table 3).

**Table 3.** Subgroup analysis of the association between area socioeconomic deprivation quartile and MAKEs.

Subgroup	No. of Patients/Events	Q4 vs. Q1 Adjusted HR (95% CI)	P for Interaction
Overall	5934/1107	1.48 (1.24–1.77)	
Age			0.824
<65 years	2970/510	1.46 (1.14–1.87)	
≥65 years	2964/597	1.51 (1.19–1.92)	
Sex			0.451
Male	3708/705	1.42 (1.14–1.77)	
Female	2226/402	1.58 (1.20–2.08)	
Admission eGFR (mL/min/1.73 m²)			0.236
<90	4451/922	1.54 (1.27–1.87)	
≥90	1483/185	1.28 (0.88–1.86)	
AKI Stage			0.380
Stage 1–2	4747/832	1.44 (1.18–1.76)	
Stage 3	1187/275	1.62 (1.18–2.22)	
AKI Type			0.112
Community Acquired	3560/742	1.55 (1.26–1.91)	
Hospital Acquired	2374/365	1.33 (1.00–1.77)	
Health Insurance Type			0.032
Employee Medical Insurance/Government- Sponsored Insurance	2540/385	1.25 (0.95–1.64)	
Resident Medical Insurance/Self-Pay	3394/722	1.63 (1.33–2.00)	

**Abbreviations:** MAKEs, Major Adverse Kidney Events; HR, hazard ratio; CI, confidence interval.

**Note:** Adjusted hazard ratios (95% CI) for the Q4 vs. Q1 comparison within each subgroup are shown. The P for interaction was calculated to test for effect modification across subgroups, with a significant interaction observed for health insurance type (*p* = 0.032).

### 3.4. Mediation analysis

**Figure 3.**
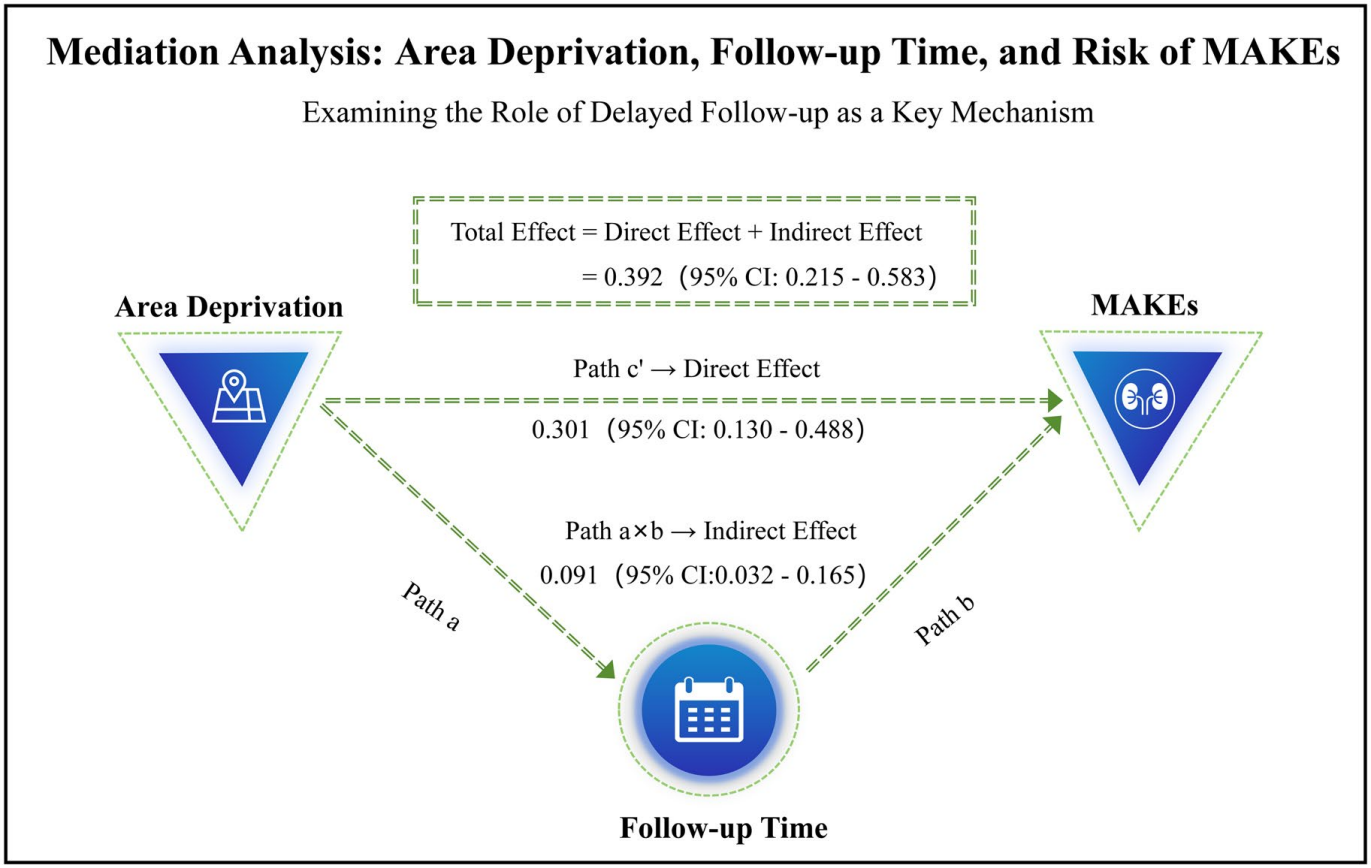
Mediation analysis of follow-up time in the association between area socioeconomic deprivation and MAKEs risk. Note: This figure presents the causal pathway model examining whether delayed follow-up time mediates the relationship between higher area socioeconomic deprivation and an increased risk of Major Adverse Kidney Events (MAKEs). The model shows that area socioeconomic deprivation (exposure) is significantly associated with a reduction in follow-up time (mediator, Path a). A shorter follow-up time is, in turn, associated with an increased risk of MAKEs (outcome, Path b). The total effect of area socioeconomic deprivation on MAKEs (c) is decomposed into a direct effect (c’) and an indirect effect (a × b). The mediation analysis indicates that a significant portion of the total effect (0.392, 95% CI: 0.215–0.583) is mediated through delayed follow-up time (indirect effect: 0.091, 95% CI: 0.032–0.165), supporting its role as a key mechanism. All effect estimates are presented with 95% confidence intervals.

The average time from AKI diagnosis to hospital discharge was 13.7 ± 10.3 days. Among patients who attended follow-up, the average time from AKI diagnosis to the first post-discharge visit was 28.6 ± 18.2 days. Mediation analysis revealed that the total effect of area socioeconomic deprivation on MAKEs was 0.392 (95% CI: 0.215–0.583, *p* < 0.001). After controlling for post-discharge follow-up duration as a mediating variable, the direct effect of area socioeconomic deprivation on MAKEs was 0.301 (95% CI: 0.130–0.488, *p* = 0.001), which remained statistically significant. The indirect effect, representing the mediating effect, was 0.091 (95% CI: 0.032–0.165, *p* = 0.002), indicating statistical significance. Calculations revealed that the proportion of variance in the association between area socioeconomic deprivation and MAKEs mediated by post-discharge follow-up time was 23.2% (95% CI: 10.5%–42.1%) (Figure 3) (Table 4).

**Table 4.** Mediation analysis of the role of post-discharge follow-up time in the association between area socioeconomic deprivation quartile and MAKEs.

Effect Decomposition	Estimate value	95% confidence interval	P value
Total Effect	0.392	(0.215, 0.583)	<0.001
Direct Effect	0.301	(0.130, 0.488)	0.001
Indirect Effect (Mediated)	0.091	(0.032, 0.165)	0.002
Proportion Mediated	23.2%	(10.5%, 42.1%)	--

**Abbreviations:** MAKEs, Major Adverse Kidney Events.

**Note:** The analysis examined whether delayed time to first post-discharge follow-up mediated the association between area socioeconomic deprivation and MAKEs risk. The indirect effect (0.091) represents the portion of the total effect explained by this pathway, accounting for 23.2% of the association. Regression-based causal mediation analysis with bootstrapping was used, adjusting for age, sex, and Charlson Comorbidity Index.

### 3.5. Sensitivity analysis

In the sensitivity analysis, the restricted mean survival time difference (RMSTD) was first used as an alternative measure. The results revealed that during the 90-day follow-up period, patients in the highest deprivation quartile (Q4) had a MAKE-free survival time that was, on average, 4.2 days shorter than that in the lowest quartile (Q1) (95% CI: 2.1–6.3 days). Second, incorporating the area socioeconomic deprivation index as a continuous variable into the model revealed that a one standard-deviation increase in the index was associated with a 15% greater risk of MAKEs (HR 1.15, 95% CI: 1.08–1.22). All sensitivity analysis results were consistent with those of the primary analysis, indicating the high robustness of the main findings of this study (Table 5).

**Table 5.** Sensitivity analyses of the association between area socioeconomic deprivation quartile and MAKEs (reference: Q1 group).

Sensitivity Analysis	Comparison	Effect Estimate (95% CI)	Conclusion
Primary Analysis (Cox Model)	Q4 vs. Q1	HR = 1.48 (1.24–1.77)	Benchmark Result
RMSTD Analysis	Q4 vs. Q1	RMSTD = −4.2 days (−6.3, −2.1)	Findings Consistent
Continuous Variable Analysis	Per 1 SD Increase	HR = 1.15 (1.08–1.22)	Findings Consistent

**Abbreviations:** MAKEs, Major Adverse Kidney Events; HR, hazard ratio; CI, confidence interval; RMSTD, Restricted Mean Survival Time Difference; SD, standard deviation.

**Note:** Sensitivity analyses were conducted to test the robustness of the primary findings: (1) Using RMSTD to estimate the absolute difference in event-free survival time; (2) Treating the deprivation index as a continuous variable to assess a dose-response relationship. The consistent results across methods support the reliability of the main association.

## 4. Discussion

This study systematically examined the independent impact of area macrolevel social environment on the long-term outcomes of AKI patients through an analysis of data from a nationwide, single-center cohort. The findings reveal that even under standardized diagnostic and treatment conditions, after fully accounting for patient characteristics, socioeconomic status, and clinical severity, patients residing in high-deprivation areas exhibited a significantly elevated risk of major MAKEs post-discharge. This risk demonstrated a marked gradient increase with increasing area socioeconomic deprivation index. This finding extends the focus of health social determinants from the traditional individual level to the macrolevel area environment, suggesting that the latter may play a significant and independent role in disease recovery.

The area socioeconomic deprivation index employed in this study was developed within the specific context of available Chinese county-level data, prioritizing the dimensions of economic capacity and healthcare resources. This focused approach aligns conceptually with the framework of validated, multi-domain indices such as the Social Deprivation Index (SDI), a composite measure whose associations with adverse health outcomes, including mortality and avoidable hospitalizations, are well established [17]. However, by concentrating on the two dimensions most directly constitutive of healthcare access and health resilience, our index provides a more streamlined and context-specific operationalization. This precision may enhance the detection of deprivation gradients specifically relevant to acute care transitions and post-discharge recovery, thereby complementing the broader societal assessment provided by more expansive indices.

The underlying mechanisms that may link area socioeconomic deprivation to poor AKI outcomes remain poorly understood. Our mediation analysis indicates that delayed first post-discharge follow-up is a key factor, accounting for approximately 23.2% of the total effect. This finding aligns with the theory of continuity of care, which emphasizes the importance of post-discharge care transitions in chronic disease management [18]. Specifically, patients in high-deprivation areas may struggle to secure timely follow-up during this critical window because of structural barriers such as transportation difficulties and financial pressures, coupled with individual factors such as relatively low health literacy. Notably, prior research has indicated that follow-up within 7 days post-discharge for AKI patients reduces readmission risk by 30% while enhancing self-efficacy and quality of life among those with comorbidities [19–21]. This contrasts sharply with the follow-up delays caused by area socioeconomic deprivation in our study, highlighting the potential influence of structural factors on healthcare accessibility. Furthermore, the remaining approximately 76.8% of the effect suggests that other important mechanisms are at play. We hypothesize that disparities in the quality of care received during hospitalization may play a significant role. This is supported by evidence showing that lower socioeconomic status is not only associated with a lower likelihood of being prescribed guideline-recommended pharmacotherapy [22]. but also with substantial challenges in medication adherence among those who do receive prescriptions [23]. Furthermore, the biological mechanisms of chronic stress warrant attention. Previous research has indicated that prolonged socioeconomic disadvantage may adversely affect renal repair by inducing chronic inflammation and sustained sympathetic nervous system activation [24,25]. Crucially, the significant interactions observed across health insurance subgroups reveal a “double-whammy” effect, demonstrating that macrolevel environmental disadvantage synergizes with an individual’s lack of protection to further exacerbate health inequities. This finding offers new insights into the complex interplay of health social determinants.

These findings align with the theoretical framework of health social determinants while providing stronger evidence through a more rigorous design. Previous studies were often limited to single regions or failed to adequately control for hospital-level confounders. Fan et al.’s [26] single-center study controlled for institutional variation but had a limited sample size and identified an association only between insurance status and ICU mortality in patients with sepsis-related AKI. In contrast, this study, which is based on a nationwide sample within a standardized clinical care system, employs a robust multivariable adjustment model to demonstrate for the first time an independent association between area socioeconomic deprivation and MAKE risk while controlling for hospital-level confounders. This methodological approach not only strengthens the evidence for considering area environment as a social determinant of health but also provides a robust model for investigating the association between macrolevel environmental factors and individual health outcomes.

The quantitative findings reveal a strong, independent association of area socioeconomic deprivation on AKI prognosis, offering clear directions for clinical practice and public health interventions. The findings indicate that patients in the most deprived areas face a 48% increased risk of MAKEs—a risk increase surpassing some established clinical risk factors [27–29], underscoring its critical importance. Additionally, mediation analysis suggests that approximately one-fourth of this effect could be mitigated through improved post-discharge follow-up, identifying a precise target for targeted interventions. This is supported by a recent meta-analysis, which found that timely post-discharge outpatient follow-up was associated with a 32% reduction in the risk of 30-day all-cause readmission, underscoring its critical role in mitigating adverse outcomes following hospitalization [30]. Therefore, in clinical practice, the area socioeconomic deprivation index should be incorporated into discharge risk assessment systems. For patients from highly deprived areas, enhanced follow-up protocols, such as proactive appointment scheduling, should be considered. At the policy level, this study suggests the need for multidimensional intervention strategies. On the one hand, the area allocation of healthcare resources should be optimized. A 2024 study by Chinese scholars revealed significant inequities in China’s healthcare workforce distribution, particularly along geographic lines. Eastern and central regions exhibit relative resource concentration or even surplus, whereas western regions face widespread shortages. Although population-based distribution equity has improved, substantial geographic disparities persist across regions [31]. On the other hand, the healthcare security system requires refinement, particularly in expanding coverage for vulnerable groups. Data from two additional Chinese studies indicate substantial disparities in reimbursement rates, covered services, and benefit levels across different insurance types. Among participants in the New Rural Cooperative Medical Scheme, Urban Employee Basic Medical Insurance, and Urban Resident Basic Medical Insurance, hospitalization rates were 17.7%, 24.2%, and 24.9% [32,33], respectively, In studies from Colombia, Mexico, and the United States, individuals enrolled in formal insurance programs had diabetes diagnosis rates that were 3.31 times, 1.47 times, and 2.23 times higher than those of uninsured individuals, respectively [9,10]. These findings demonstrate the correlation between health insurance category and residents’ access to medical resources. These measures resonate with the “double whammy” effect identified in this study, underscoring the need for comprehensive interventions.

However, this study also has several limitations that merit consideration. First, its retrospective observational design inherently precludes the establishment of causality. Although we controlled for confounders through multivariable adjustments and series analysis, unmeasured confounders, such as patient health literacy and social support levels, may still exist. Second, the outcome data relied on our hospital’s electronic medical record system, potentially insufficiently capturing post-discharge care at other facilities, mortality, or readmissions. Although supplemented by CDC mortality data, information gaps may persist. Third, while the area socioeconomic deprivation index was constructed on the basis of classical theory and data availability, it excluded potentially important dimensions such as environmental quality and educational attainment, potentially limiting the comprehensiveness of the deprivation assessment. Fourth, our identification of AKI relied solely on serial serum creatinine measurements without urine output criteria, potentially leading to under-ascertainment, particularly of non-oliguric cases. This may limit the generalizability of our findings to the broader AKI spectrum, though the association observed within this stringently defined cohort remains robust. Fifth, as a single-center study conducted in a large tertiary national medical center, the generalizability of our findings may be limited. Patients admitted to our center might not be fully representative of the broader AKI population treated in community or regional hospitals, particularly in terms of disease complexity and access to pre-referral care.

This study identifies a significant association: area socioeconomic deprivation is a robust and independent marker associated with worse outcomes in AKI patients. These findings highlight the observed association of macrolevel social environment with patient prognosis and suggest a need to consider integrating assessments of social context into holistic care models to address health equity. However, given the observational design, this association warrants further investigation in prospective studies to establish causality. The results underscore the importance of research that bridges clinical care and social determinants of health.

## Ethics approval and consent to participate

This study was approved by the Institutional Review Board (IRB) of the General Hospital of the PLA (Lun Zi, No. S2025-260-01). The requirement for informed consent was waived because all personally identifiable information of enrolled patients was anonymized prior to analysis. The study strictly adhered to the principles of the Declaration of Helsinki.

## Supplementary Material

Supplemental Material

## Data Availability

The data that support the findings of this study are available from the Medical Innovation Research Division, Chinese PLA General Hospital, but there are restrictions regarding the availability of these data, which were used under license for the present study and are not publicly available. However, the data are available from the corresponding author (Zhe Feng) upon reasonable request and with the permission of the Medical Innovation Research Division.

## Abbreviations

AKI: Acute Kidney Injury
CDC: Center for Disease Control and Prevention
CI: Confidence Interval
CKD: Chronic Kidney Disease
eGFR: estimated Glomerular Filtration Rate
HR: Hazard Ratio
ICU: Intensive Care Unit
KDIGO: Kidney DiseaseImproving Global Outcomes
MAKEs: Major Adverse Kidney Events
PCA: Principal Component Analysis
RRT: Renal Replacement Therapy
RMSTD: Restricted Mean Survival Time Difference
SD: Standard Deviation
SDI: Social Deprivation Index
VIF: Variance Inflation Factor

## References

[1] Susantitaphong P, Cruz DN, Cerda J, et al. World incidence of AKI: a meta-analysis. Clin J Am Soc Nephrol. 2013;8(9):1482–1493. doi: 10.2215/CJN.00710113. 23744003 PMC3805065

[2] Chertow GM, Burdick E, Honour M, et al. Acute kidney injury, mortality, length of stay, and costs in hospitalized patients. J Am Soc Nephrol. 2005;16:3365–3370. doi: 10.1681/ASN.2004090740. 16177006

[3] Hoste EAJ, Kellum JA, Selby NM, et al. Global epidemiology and outcomes of acute kidney injury. Nat Rev Nephrol. 2018;14(10):607–625. doi: 10.1038/s41581-018-0052-0. 30135570

[4] James MT, Bhatt M, Pannu N, et al. Long-term outcomes of acute kidney injury and strategies for improved care. Nat Rev Nephrol. 2020;16(4):193–205. doi: 10.1038/s41581-019-0247-z. 32051567

[5] Chaïbi K, Ehooman F, Pons B, et al. Long-term outcomes after severe acute kidney injury in critically ill patients: the SALTO study. Ann Intensive Care. 2023;13(1):18. doi: 10.1186/s13613-023-01108-x. 36907976 PMC10008759

[6] Neyra JA, Chawla LS. Acute kidney disease to chronic kidney disease. Crit Care Clin. 2021;37(2):453–474. doi: 10.1016/j.ccc.2020.11.013. 33752866

[7] Sadabadi F, Talkhi N, Omouri-Kharashtomi M, et al. Association between socio-economic status (ses) and the traditional risk factors of cardiovascular diseases (CVD): a cross-sectional MASHAD cohort study results. Health Sci Rep. 2025;8(5):e70721. doi: 10.1002/hsr2.70721. 40309617 PMC12040752

[8] Akinbule OO, Tor-Anyiin FN, Adenusi SA. Impact of socio-economic factors and lifestyle behaviours on the quality of life of diabetes patients in Markurdi Benue state Nigeria. Sci Rep. 2025;15(1):34498. doi: 10.1038/s41598-025-15889-7. 41044069 PMC12494918

[9] Kim TJ, Vonneilich N, Lüdecke D, et al. Income, financial barriers to health care and public health expenditure: a multilevel analysis of 28 countries. Soc Sci Med. 2017;176:158–165. doi: 10.1016/j.socscimed.2017.01.044. 28153752

[10] Gakidou E, Mallinger L, Abbott-Klafter J, et al. Management of diabetes and associated cardiovascular risk factors in seven countries: a comparison of data from national health examination surveys. Bull World Health Organ. 2011;89(3):172–183. doi: 10.2471/BLT.10.080820. 21379413 PMC3044248

[11] Srisawat N, Kulvichit W, Mahamitra N, et al. The epidemiology and characteristics of acute kidney injury in the Southeast Asia intensive care unit: a prospective multicentre study. Nephrol Dial Transplant. 2020;35(10):1729–1738. doi: 10.1093/ndt/gfz087. 31075172

[12] Holmes J, Phillips D, Donovan K, et al. Acute kidney injury, age, and socioeconomic deprivation: evaluation of a national data set. Kidney Int Rep. 2019;4(6):824–832. doi: 10.1016/j.ekir.2019.03.009. 31194105 PMC6551509

[13] Ostermann M, Bellomo R, Burdmann EA, et al. Controversies in acute kidney injury: conclusions from a kidney disease: improving Global Outcomes (KDIGO) Conference. Kidney Int. 2020;98(2):294–309. doi: 10.1016/j.kint.2020.04.020. 32709292 PMC8481001

[14] Xu X, Nie S, Liu Z, et al. Epidemiology and clinical correlates of AKI in Chinese hospitalized adults. Clin J Am Soc Nephrol. 2015;10(9):1510–1518. doi: 10.2215/CJN.02140215. 26231194 PMC4559507

[15] Quan H, Li B, Couris CM, et al. Updating and validating the Charlson comorbidity index and score for risk adjustment in hospital discharge abstracts using data from 6 countries. Am J Epidemiol. 2011;173(6):676–682. doi: 10.1093/aje/kwq433. 21330339

[16] Miller WG, Kaufman HW, Levey AS, et al. National Kidney Foundation Laboratory Engagement Working Group recommendations for implementing the ckd-epi 2021 race-free equations for estimated glomerular filtration rate: practical guidance for clinical laboratories. Clin Chem. 2022;68(4):511–520. doi: 10.1093/clinchem/hvab278. 34918062

[17] Butler DC, Petterson S, Phillips RL, et al. Measures of social deprivation that predict health care access and need within a rational area of primary care service delivery. Health Serv Res. 2013;48(2 Pt 1):539–559. doi: 10.1111/j.1475-6773.2012.01449.x. 22816561 PMC3626349

[18] Shin H. A survey of the working status of family medicine physicians in clinics and hospitals in Korea. BMC Fam Pract. 2020;21(1):82. doi: 10.1186/s12875-020-01154-5. 32384873 PMC7323610

[19] Kucharczuk C, Lightheart E, Kodan A, et al. Standardized discharge planning tool leads to earlier discharges and fewer readmissions. J Nurs Care Qual. 2022;37(1):54–60. doi: 10.1097/NCQ.0000000000000558. 33734187

[20] Hu R, Gu B, Tan Q, et al. The effects of a transitional care program on discharge readiness, transitional care quality, health services utilization and satisfaction among Chinese kidney transplant recipients: a randomized controlled trial. Int J Nurs Stud. 2020;110:103700. doi: 10.1016/j.ijnurstu.2020.103700. 32739670

[21] Lin S, Xiao LD, Chamberlain D, et al. Nurse-led health coaching programme to improve hospital-to-home transitional care for stroke survivors: a randomised controlled trial. Patient Educ Couns. 2022;105(4):917–925. doi: 10.1016/j.pec.2021.07.020. 34294494

[22] Forbes AK, Hinton W, Feher MD, et al. Implementation of chronic kidney disease guidelines for sodium-glucose co-transporter-2 inhibitor use in primary care in the UK: a cross-sectional study. EClinicalMedicine. 2024;68:102426. doi: 10.1016/j.eclinm.2024.102426. 38304744 PMC10831804

[23] Hofer R, Choi H, Mase R, et al. Mediators and moderators of improvements in medication adherence. Health Educ Behav. 2017;44(2):285–296. doi: 10.1177/1090198116656331. 27417502 PMC5237412

[24] Stoecker JB, Cohen JB, Belkin N, et al. The association between socioeconomic factors and incident peripheral artery disease in the chronic renal insufficiency cohort (CRIC). Ann Vasc Surg. 2022;80:196–205. doi: 10.1016/j.avsg.2021.07.057. 34656710 PMC8977117

[25] Saunders MR, Ricardo AC, Chen J, et al. Area socioeconomic status and risk of hospitalization in patients with chronic kidney disease: a chronic renal insufficiency cohort study. Medicine (Baltimore). 2020;99(28):e21028. 32664108 10.1097/MD.0000000000021028 PMC7360239

[26] Fan H, Zhao Y, Chen GD, et al. Health insurance status and risk factors of mortality in patients with septic acute kidney injury in Ningbo, China. J Int Med Res. 2019;47(1):370–376. doi: 10.1177/0300060518802526. 30328368 PMC6384465

[27] Pan H-C, Wu P-C, Wu V-C, et al. A nationwide survey of clinical characteristics, management, and outcomes of acute kidney injury (AKI) – patients with and without preexisting chronic kidney disease have different prognoses. Medicine (Baltimore). 2016;95(39):e4987. doi: 10.1097/MD.0000000000004987. 27684854 PMC5265947

[28] Hu L, Gao L, Zhang D, et al. The incidence, risk factors and outcomes of acute kidney injury in critically ill patients undergoing emergency surgery: a prospective observational study. BMC Nephrol. 2022;23(1):42. doi: 10.1186/s12882-022-02675-0. 35065624 PMC8782702

[29] Zhang M, Zeng J, Ge Y, et al. Risk factors and prognosis of post-surgical acute kidney injury in elderly patients based on the MIMIC-IV database. Eur J Med Res. 2025;30(1):491. doi: 10.1186/s40001-025-02762-6. 40537854 PMC12178035

[30] Balasubramanian I, Andres EB, Malhotra C. Outpatient follow-up and 30-day readmissions: a systematic review and meta-analysis. JAMA Netw Open. 2025;8(11):e2541272. doi: 10.1001/jamanetworkopen.2025.41272. 41186947 PMC12587199

[31] Chai Y, Xian G, Kou R, et al. Equity and trends in the allocation of health human resources in China from 2012 to 2021. Arch Public Health. 2024;82(1):175. doi: 10.1186/s13690-024-01407-0. 39375748 PMC11460223

[32] Chen R, Li N, Liu X. Study on the equity of medical services utilization for elderly enrolled in different basic social medical insurance systems in an underdeveloped city of Southwest China. Int J Equity Health. 2018;17(1):54. doi: 10.1186/s12939-018-0765-5. 29716603 PMC5930827

[33] Zhang F, Shi X, Zhou Y. The impact of health insurance on healthcare utilization by migrant workers in China. Int J Environ Res Public Health. 2020;17(6):1852. doi: 10.3390/ijerph17061852. PMC7143864 32178431

