# Supplementary material for: Impact of area socioeconomic deprivation on major adverse renal events in patients with acute kidney injury: a retrospective cohort study of a single-center national patient population
Source: Ann Med. 2026 Jun 4;58(1):2674449. doi: 10.1080/07853890.2026.2674449 (PMC13237789; doi:10.1080/07853890.2026.2674449)

**Supplementary Table S1. Principal component analysis for the construction of the Area Socioeconomic Deprivation Index**

| Dimension | Indicator | Component Loading |
| --- | --- | --- |
| Economic Dimension | Per capita GDP | 0.92 |
|  | Per capita disposable income | 0.90 |
|  | General public budget revenue | 0.88 |
| Healthcare Resource Dimension | number of licensed physicians per thousand population | 0.79 |
|  | number of healthcare institution beds per thousand population | 0.73 |
|  | PCA Result for Component 1 (Extracted) |  |
|  | Eigenvalue | 3.27 |
|  | Variance Explained (%) | 65.3% |

**Note:** Principal component analysis (PCA) was applied to construct the Area Socioeconomic Deprivation Index. Five county-level indicators, standardized using Z-scores, were reduced to a single component (eigenvalue = 3.27), explaining 65.3% of the total variance. All indicator loadings were high (0.71–0.92), confirming strong representation of the underlying socioeconomic and healthcare resource dimensions. A higher composite score indicates lower area deprivation. The Kaiser–Meyer–Olkin measure (0.82) and Bartlett’s test of sphericity (P < 0.001) confirmed the suitability of the data for PCA.

Figure S1

Flowchart of AKI identification and classification

Note: This diagram outlines the algorithm for detecting AKI episodes based on KDIGO serum creatinine criteria and classifying them as community- or hospital-acquired from inpatient data.


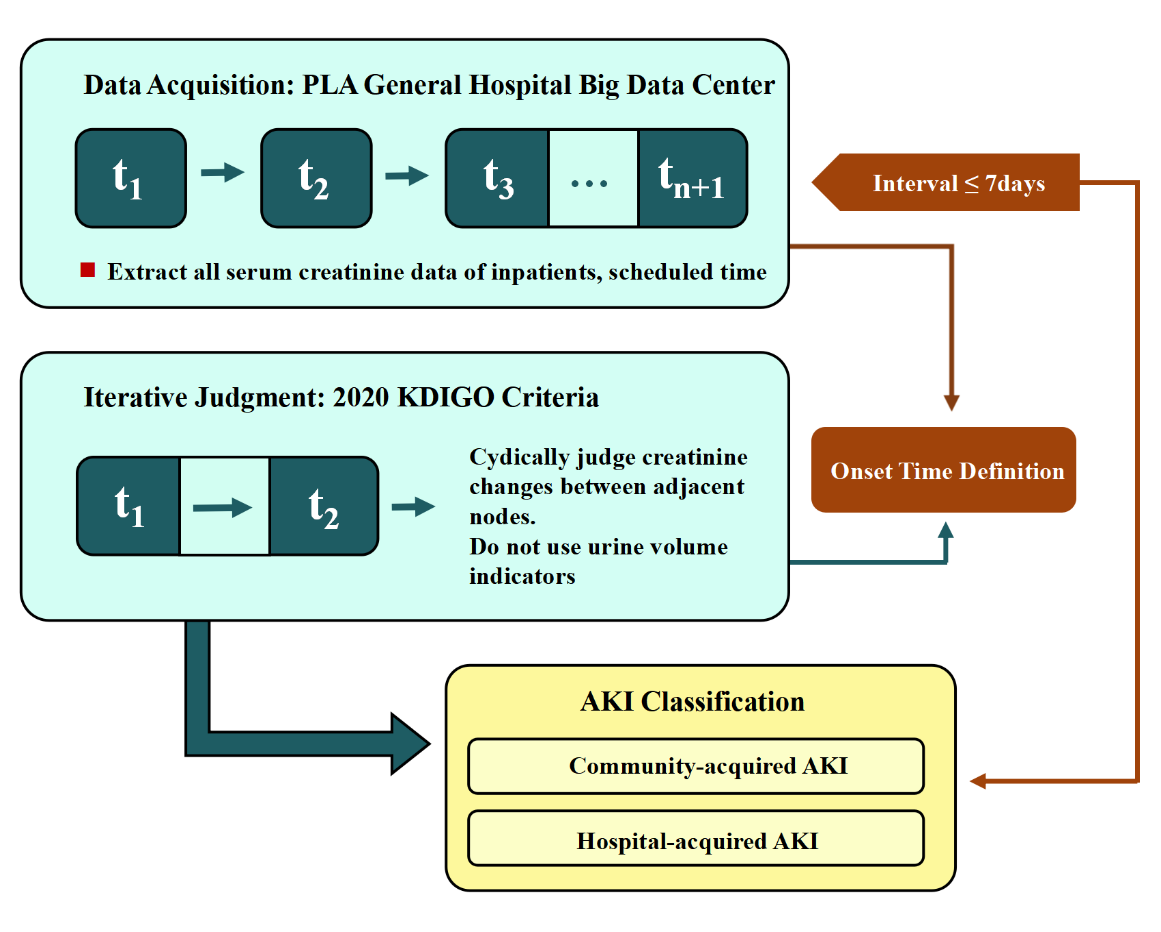

Supplement: Supplemental Material [file IANN_A_2674449_SM1281.docx]
